# High purpose in life of older adults is associated with younger age, higher education, and being female

**DOI:** 10.1055/s-0046-1818610

**Published:** 2026-04-07

**Authors:** Sabrina Aparecida da Silva, Gabriela dos Santos, Tiago Nascimento Ordonez, Wellington Lourenço Oliveira, Diana dos Santos Bacelar, Maria Antonia Antunes de Souza, Laydiane Alves Costa, Henrique Salmazo da Silva, Beatriz Aparecida Ozello Gutierrez, Rosa Yuka Sato Chubaci, Sonia Maria Dozzi Brucki, Thais Bento Lima da Silva

**Affiliations:** 1 Universidade de São Paulo, Escola de Artes, Ciências e Humanidades, Gerontologia, São Paulo SP, Brazil.; 2 Universidade Federal do Recôncavo da Bahia, Centro de Ciências da Saúde, Santo Antônio de Jesus BA, Brazil.; 3 Universidade de São Paulo, Faculdade de Medicina, Hospital das Clínicas, Departamento de Neurologia, São Paulo SP, Brazil.

**Keywords:** Sociodemographic Factors, Goals, Aged, Personal Satisfaction

## Abstract

**Background:**

Purpose in life (PiL) is an important aspect of psychological wellbeing, which can be influenced by sociodemographic factors.

**Objective:**

To analyze the relationship between sociodemographic variables and PiL in older individuals.

**Methods:**

A quantitative study that evaluated 189 participants using the PiL Scale (PLS) was conducted. Participants were divided into two groups according to median PLS score. The Mann-Whitney U-test was used to assess group differences, given the non-normal distribution of variables. Spearman's bivariate correlations and a stepwise logistic regression model were applied to identify predictors of PiL.

**Results:**

Of the total participants, 77.25% were female. The mean age was significantly lower in the high PiL group ( *p* = 0.017). Spearman's correlations revealed a weak negative relationship between age and PiL (rho = −0.151; *p* = .038) and a weak positive relationship between schooling and PiL (rho = 0.156; *p* = 0.032). Logistic regression indicated that higher education (OR = 1.087; *p* = 0.017) and being female (OR = 2.546; *p* = 0.018) were associated with higher PiL.

**Conclusion:**

High PiL was associated with higher education and being female. Although age showed a negative correlation, the variable did not remain an independent predictor in the final multivariate model.

## INTRODUCTION

Purpose in life (PiL) is a psychological construct that reflects an individual's sense of meaning, direction, and intention in life, guiding how goals and objectives are defined and pursued. 1 According to Ryff, 2 PiL is one of the six domains of psychological wellbeing, together with self-acceptance, positive relations, autonomy, environmental mastery, and personal growth.

Higher PiL among older adults has been associated with sociodemographic and biopsychosocial factors, including employment status, marital status, functional competence in activities of daily living, quality of interpersonal relationships, health, higher socioeconomic and schooling levels, and religiosity. 3 These factors contribute to better living conditions, greater access to resources, and increased opportunities for leisure and social engagement.

Coelho et al. 4 investigated biopsychosocial correlates of PiL in 1,330 Portuguese adults aged 55 to 84 years, using sociodemographic data and multiple psychological instruments. The results indicated that schooling, stress, spirituality, optimism, social support, and physical health-related quality of life were positively associated with PiL.

Using a machine learning approach, Mei et al. 5 examined correlates of PiL in 1,839 individuals without dementia. Loneliness showed the strongest negative association with PiL, whereas perceived social support, social participation, and engagement in cognitively stimulating activities during midlife were associated with higher PiL.

Evidence indicates that individuals with higher PiL attribute greater meaning to life experiences and show better health outcomes. 1 Purpose in life has been identified as a protective factor against neurodegenerative conditions, including Alzheimer's disease, and against depression, low self-esteem, and stress. 6

Sutin et al. 7 reported that middle-aged and older adults with higher PiL performed better on episodic memory and verbal fluency tasks and had a more positive perception of their memory, with these benefits remaining stable for over a decade. 8 These associations were consistent across age, sex, race/ethnicity, schooling, and income, and included lower risk of dementia, motoric cognitive risk syndrome, and Behavioral and Psychological Symptoms of Dementia (BPSD), as well as greater emotional resilience and social engagement.

A systematic review by AshaRani et al. 9 identified female sex, higher education and income, marital status, health, inner strength, and social integration as key correlates of PiL. Higher PiL was associated with lower mortality, reduced cognitive decline, greater social participation, and healthier behaviors.

Similarly, Stewart et al. 10 found that PiL supported decision-making in 1,081 community-dwelling older adults without dementia, particularly among those with lower cognitive performance. Younger age, male sex, and higher education were also related to better decision-making.

Despite extensive international evidence, studies examining PiL in Brazil remain scarce. This gap limits understanding of aging and well-being in a context characterized by social inequality and restricted access to health and educational resources. Therefore, the present study aimed to analyze the relationship between sociodemographic variables and PiL among cognitively healthy older adults in Brazil, contributing evidence from a low- and middle-income country.

## METHODS

### Study design and participants

The present descriptive cross-sectional study used data collected during the 24-month follow-up assessment (T4) of a randomized controlled clinical trial conducted between April and May 2024. The parent trial investigated the effectiveness of a multifactorial cognitive stimulation program on cognitive and psychosocial outcomes in cognitively healthy older adults. The intervention lasted 18 months, with assessments performed in person at baseline and throughout the intervention period, followed by a post-intervention follow-up at 24 months.

For the present analysis, data from 189 older adults who completed the follow-up assessment were included, regardless of original trial group allocation. Participants were initially recruited from hospital-run Community Centers for Older Adults, and from Pensioner and Retiree Associations, in São Paulo, Brazil. While all assessments in the parent trial were conducted in person, data for the present cross-sectional study were collected by telephone.

Although participants had previously taken part in a cognitive stimulation intervention, the present analysis was conducted after completion of the intervention phase. Importantly, the intervention was not specifically designed to target existential or motivational constructs such as PiL. Moreover, inclusion of participants from all original trial arms reduces the likelihood that trial-related selection factors systematically influenced PiL in the present cross-sectional analysis.

At the initial screening of the clinical trial, individuals aged ≥ 60 years with sufficient comprehension to perform the required tasks were eligible. All participants completed the Brazilian version of the Mini-Mental State Examination for telephone application (Braztel-MMSE) 11 and the Geriatric Depression Scale (GDS). 12 13 Individuals with scores suggestive of dementia on the Braztel-MMSE or scores > 5 on the GDS were excluded. Additional exclusion criteria included sensory or motor impairments that could hinder task performance, severe psychiatric disorders, clinical or neuroimaging evidence of cerebrovascular disease, and a diagnosis of dementia.

### Ethical aspects

The study was approved by the Ethics Committee for Research in Humans of Hospital das Clínicas da Faculdade de Medicina da Universidade de São Paulo, under permit no. 4.357.429 (CAAE: 35462620.2.0000.0068).

### Data collection

The sociodemographic variables were assessed using a structured questionnaire collecting information on sex, age, schooling, marital status, and employment status (retired, pensioner, or otherwise). Level of PiL was measured using the PiL subscale (PLS) from the Ryff and Keyes' Psychological Well-Being scales, translated and validated for use in Brazil by Cristovão. 14 The original validation study was conducted via in-person administration, whereas, for the present study, the PLS was administered by telephone, with standardized instructions read verbatim to participants.

The PLS is a 10-item scale assessing respondents' agreement level with statements related to PiL. To calculate the final score, items 2, 3, 5, 6, and 10 are reverse-coded, and item scores (ranging from 1–6) are averaged, with higher scores indicating greater PiL.

### Data analysis

The data collected were analyzed descriptively and participants categorized into 2 groups based on median score on the PLS. Group 1 (G1) included participants scoring ≤ 4.10, whereas group 2 (G2) comprised those scoring > 4.10, using the median as the cut-off for categorization.

Given the non-normal distribution of the continuous and ordinal variables, the Mann-Whitney U-test was applied to compare differences between the groups. In addition, bi-variate Spearman correlation was performed among numeric variables, while multivariate logistic regression analysis (step-wise model) was conducted to identify variables predicting PiL.

Lastly, all statistical analyses were conducted using the JASP software package (0.95.3.0) (open source, University of Amsterdam). The significance level (α) was set at 0.05 for all analyses.

## RESULTS

Of the total sample, 146 (77.25%) participants were female. Group 1 had a mean age of 70.17 (±4.93) years and G2 a mean age of 68.50 (±4.76) years, representing a statistically significant difference between the 2 groups ( *p* = 0.017). Overall, mean schooling was 17.64 (±4.68) years, with no statistically significant difference between the groups. Respondents were predominantly married (46.57%) and retired (93.65%), with no group differences for these variables. Median score on the PLS was 3.80 (minimum = 2.70; maximum = 4.10) for G1 and 4.40 (minimum 4.20; maximum 4.90) for G2, constituting a statistically significant group difference ( *p* < 0.001) ( Table 1 ).

**Table 1:** Sociodemographic and psychosocial characteristics of total sample

Variable		Total		G1 (PL ≤ Median)		G2 (PL > Median)		*p-value*
		n	%	n	%	n	%	
Sex	Female	146	77.25	76	72.38	70	83.33	0.074 a
	Male	43	22.75	29	27.62	14	16.67	
Age (years)	Mean ± SD	69.43 ± 4.91		70.17 ± 4.93		68.50 ± 4.76		0.017 b
	Median (min–max.)	69.00 (62.00–86.00)		69.00 (62.00–85.00)		68.50 (62.00–86.00)		
Schooling (years)	Mean ± SD	17.64 ± 4.68		17.09 ± 3.71		18.33 ± 5.60		0.167 b
	Median (min–max)	17.00 (7.00–40.00)		16.00 (8.00–30.00)		18.00 (7.00–40.00)		
Marital status	Married	88	46.57	48	45.71	40	47.62	0.806 a
	Divorced	29	15.34	18	17.14	11	13.10	
	Single	43	22.75	22	20.95	21	25.00	
	Widowed	29	15.34	17	16.19	12	14.29	
Retired or pensioner	Yes	177	93.65	97	92.38	80	95.24	0.423 a
	No	12	6.35	8	7.62	4	4.76	
PLS (total score)	Mean ± SD	4.02 ± 0.50		3.66 ± 0.37		4.47 ± 0.19		< 0.001 b
	Median (min–max.)	4.10 (2.70–4.90)		3.80 (2.70–4.10)		4.40 (4.20–4.90)		

Abbreviations: Max., maximum; Min., minimum; PLS, Purpose in Life Scale; SD, standard deviation.

Notes: ^a^ Chi-squared test; ^b^ Mann-Whitney U-test.

**Figure 1:**
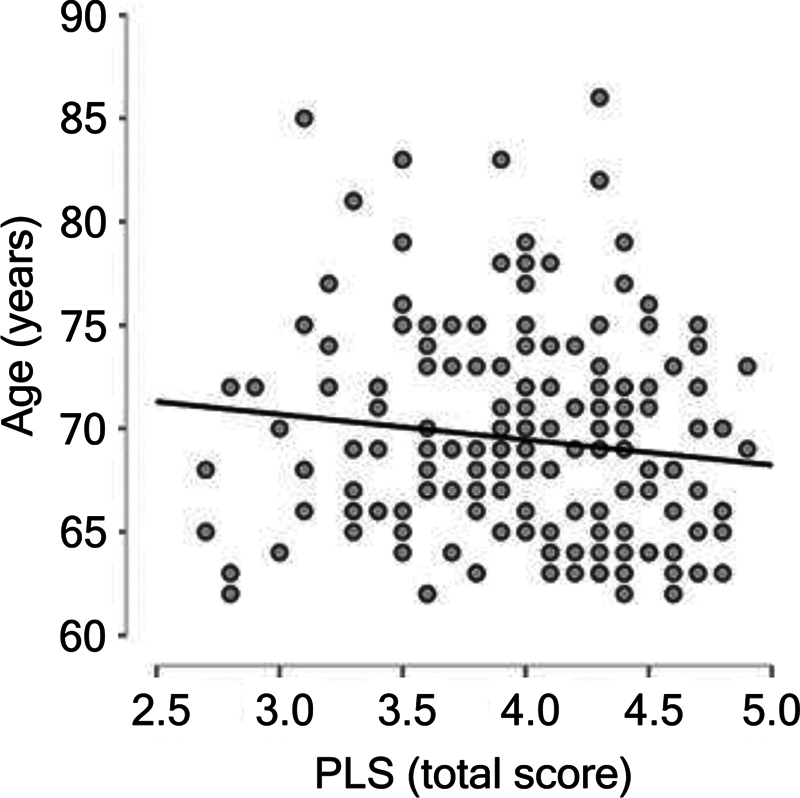
Negative Spearman bivariate correlation (rho = −0.151; *p* = 0.038) between total score on the Purpose in Life Scale (PLS) and age (in years).

**Figure 2:**
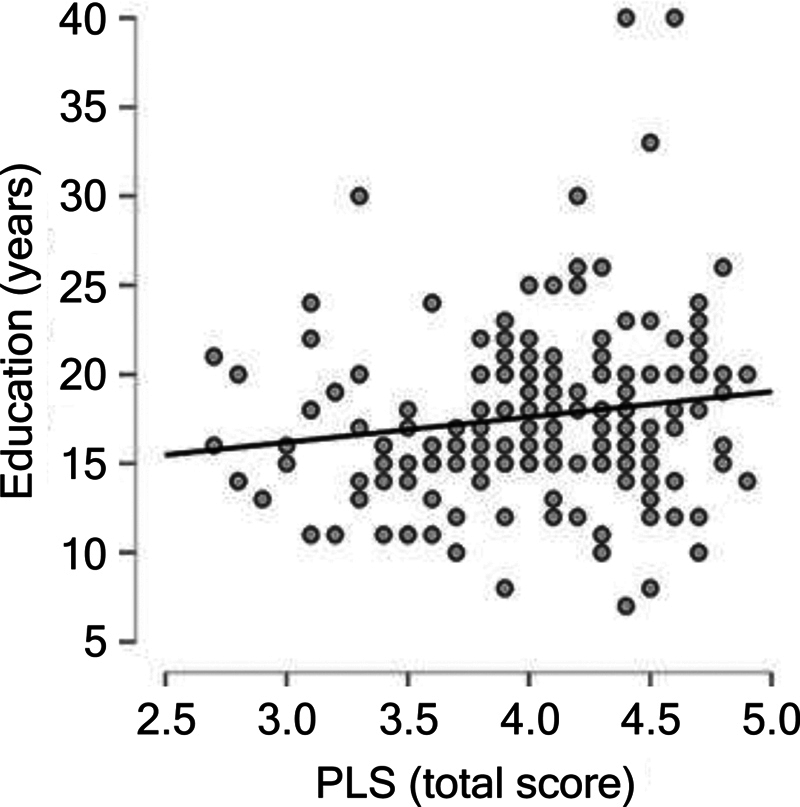
Positive Spearman bivariate correlation (rho = 0.156; *p* = 0.032) between total score on the PLS and schooling (in years).

Bivariate Spearman's correlations indicated statistically significant associations between PiL and the sociodemographic variables. A weak inverse relationship was observed between the PLS scores and age (rho = −0.151; *p* = .038), suggesting that PLS scores tend to decrease slightly with increasing age ( Figure 1 ). Conversely, a weak positive correlation was found between PLS scores and years of schooling (rho = 0.156; *p* = .032), indicating that more years of schooling are associated with slightly higher PLS scores ( Figure 2 ).

Lastly, a binary logistic regression was performed using the stepwise method to select the most relevant predictors of a high PiL from a set of sociodemographic variables. The results from the final model show that the odds of an individual having a high PiL increase by 8.7% for each additional year of schooling (b = 0.084; *p* = 0.017; OR = 1.087). Also, the odds of high PiL increase by 154.6% for female participants compared to male participants (b = 0.935; *p* = 0.018; OR = 2.546). Therefore, more years of schooling and being female were identified as significant predictors associated with a greater probability of having high PiL ( Table 2 ).

**Table 2:** Multivariate logistic regression (stepwise model)

Parameter	Estimate	SE	Odds ratio	z	Wald Statistic	df	*p*	95%Confidence interval	
								Lower bound	Upper bound
(Intercept)	−2.432	0.790	0.088	−3.079	9.482	1	0.002	0.019	0.413
Achooling (years)	0.084	0.035	1.087	2.395	5.738	1	0.017	1.015	1.164
Sex (Female)	0.935	0.395	2.546	2.367	5.600	1	0.018	1.174	5.521

Abbreviations: df, degrees of freedom; SE, standard error.

Note: PL_median level ‘1’ coded as PL > median; Estimate = beta coefficient.

## DISCUSSION

The present study investigated the relationship between sociodemographic variables and level of PiL in cognitively healthy older adults. The descriptive analysis revealed a predominance of women in the sample, which may reflect both the demographic feminization of aging and a greater tendency among women to engage in social and health-related activities, rather than a confirmed behavioral difference. 15 Data from the Brazilian Institute of Geography and Statistics 16 indicate that women accounted for 55.7% of the older population in Brazil in the 2022 Census, supporting the observed sex distribution.

The mean age of the sample was 69.4 years, possibly reflecting greater participation of younger older adults in community-based activities, such as centers and associations for retirees, from which participants were recruited. This finding is consistent with those of Luster et al., 17 whose sample had a similar mean age and included individuals engaged in regular social activities.

Married and retired individuals predominated in the sample, although these variables were not statistically associated with PiL. Retirement has been shown to influence mental and physical health in heterogeneous ways, depending on the circumstances under which it occurs. Neumann-Böhme et al. 18 reported lower life satisfaction among individuals who retired due to disability, whereas higher satisfaction was observed among those receiving lifetime pensions. These findings suggest that financial stability and social support may play an important role in wellbeing later life.

Inferential analyses indicated that PiL was associated with age, schooling, and sex. However, the associations observed were weak, indicating that sociodemographic variables explained only a limited proportion of the variance in PiL. A negative correlation was found between age and PiL, with older age associated with lower PiL scores. These results are consistent with those of previous studies 1 8 suggesting a gradual decline in PiL with aging, potentially reflecting changes in health status, social networks, and opportunities for engagement in late life. 19 Nevertheless, other studies have reported no significant moderating effect of age on PiL, highlighting heterogeneity across samples. 20

A positive association was observed between PiL and years of schooling, with higher educational attainment associated with higher PiL scores. These findings align with those of Oliveira et al., 21 who reported higher PiL and better global cognition among highly educated older adults. Individuals with more years of formal education may engage in cognitively and socially complex activities that support psychological wellbeing. 14 22 Despite this association, the effect size was small, suggesting that schooling alone is insufficient to account for meaningful differences in PiL. Lower educational attainment has been associated with reduced cognitive reserve and poorer health outcomes, which may indirectly influence PiL. 23

Consistent with previous research, higher PiL has been associated with more favorable health and psychosocial outcomes 24 However, given the modest associations observed in the present study, these findings should be interpreted with caution, as PiL is likely influenced by a broader set of determinants beyond basic sociodemographic characteristics.

Logistic regression revealed associations of PiL with schooling and sex, with men scoring lower on the PLS than women. Similar findings have been reported by Xi et al. 2 25 and may reflect sex-related social norms and differences in social engagement. Other studies suggest that lower PiL in older men may be related to mental health challenges and smaller social networks. 26 Qualitative evidence further indicates that men and women may conceptualize PiL differently, reflecting life-course experiences rather than causal effects. 27

Importantly, the present study assessed a limited set of sociodemographic variables. Other relevant determinants of PiL, such as subclinical depressive symptoms, social support, physical health, functional status, and religiosity, were not evaluated and may account for a substantial proportion of unexplained variance in PiL.

In the present sample, higher PiL was independently associated with higher level of schooling and female sex, while age showed only a weak bivariate association. These associations were weak, indicating that sociodemographic variables alone explain only a limited portion of variability in PiL.

The high level of schooling and predominance of women in the sample substantially limit the generalizability of the findings to the broader Brazilian older adult population, particularly individuals with lower levels of schooling and reduced access to social resources.

Future studies should include more heterogeneous samples and incorporate a broader range of psychosocial, health-related, and functional variables. Longitudinal designs may further contribute to understanding how PiL evolves over time and which factors most strongly influence maintenance of PiL in later life.
